# Clinical Malaria Transmission Trends and Its Association with Climatic Variables in Tubu Village, Botswana: A Retrospective Analysis

**DOI:** 10.1371/journal.pone.0139843

**Published:** 2016-03-16

**Authors:** Elijah Chirebvu, Moses John Chimbari, Barbara Ntombi Ngwenya, Benn Sartorius

**Affiliations:** 1 Okavango Research Institute, University of Botswana, Private Bag 285, Maun, Botswana; 2 College of Health Sciences, Howard Campus, University of KwaZulu-Natal, Durban 4041, South Africa; 3 Discipline of Public Health Medicine, School of Nursing and Public Health, University of KwaZulu-Natal, Durban, South Africa; Johns Hopkins University, UNITED STATES

## Abstract

Good knowledge on the interactions between climatic variables and malaria can be very useful for predicting outbreaks and preparedness interventions. We investigated clinical malaria transmission patterns and its temporal relationship with climatic variables in Tubu village, Botswana. A 5-year retrospective time series data analysis was conducted to determine the transmission patterns of clinical malaria cases at Tubu Health Post and its relationship with rainfall, flood discharge, flood extent, mean minimum, maximum and average temperatures. Data was obtained from clinical records and respective institutions for the period July 2005 to June 2010, presented graphically and analysed using the Univariate ANOVA and Pearson cross-correlation coefficient tests. Peak malaria season occurred between October and May with the highest cumulative incidence of clinical malaria cases being recorded in February. Most of the cases were individuals aged >5 years. Associations between the incidence of clinical malaria cases and several factors were strong at lag periods of 1 month; rainfall (r = 0.417), mean minimum temperature (r = 0.537), mean average temperature (r = 0.493); and at lag period of 6 months for flood extent (r = 0.467) and zero month for flood discharge (r = 0.497). The effect of mean maximum temperature was strongest at 2-month lag period (r = 0.328). Although malaria transmission patterns varied from year to year the trends were similar to those observed in sub-Saharan Africa. Age group >5 years experienced the greatest burden of clinical malaria probably due to the effects of the national malaria elimination programme. Rainfall, flood discharge and extent, mean minimum and mean average temperatures showed some correlation with the incidence of clinical malaria cases.

## Introduction

Malaria is considered to be the most important vector borne disease in tropical regions, posing a very serious public health problem in most developing countries. The World Health Organisation [1] report of March 2013 estimated that about 3.3 billion people (half of the world's population) are at risk of contracting malaria. In 2012 about 207 million cases (with an uncertainty range of 135 million to 287 million) and an estimated 627 000 deaths (with an uncertainty range of 473 000 to 789 000) were recorded [2]. Globally, there has been a decline in mortality rates of more than 42% since 2000. In the WHO African Region 49% of the reduction is attributed to increased prevention and control measures [2]. In 2010, 90% of all malaria deaths occurred in the WHO African Region, mostly among children under five years of age [3].

In many parts of Africa malaria occurs in distinct seasons and epidemics that may be caused by a range of factors such as movement and displacement of human populations, breakdown of control activities, environmental changes and climatic factors [4]). Malaria has been recognized as one of the diseases most sensitive to climate change [5, 6]. Temperature, humidity and rainfall affect the degree of malaria transmission either through changes in the duration of mosquito and parasite life cycle or through influences on human or parasite behaviour [7, 8]. Floods also increase the transmission of communicable diseases including malaria [9]. The effects of climate change on malaria have been discussed and remain a complex and controversial issue. Various studies [10–12] have argued that climate change (warming) is not responsible for observed changes in malaria transmission owing to other socioeconomic factors such as land use change, population growth, migration changes and economic development [13]. However the subject remains controversial since other studies found a link between changes in climatic factors and malaria transmission [14–23]. According to Pascual et al. [14]both theories may be correct since various factors complement and interact each other at different time scales.

Links between temperature and malaria transmission were observed in many countries including East African Highlands [24], North East Tanzania [25], Rwanda [26] and South Africa [27]. A study in Botswana [28] concluded that both rainfall and standardized annual malaria incidence anomalies from December to February were significantly related to sea surface temperatures in the eastern Pacific, meaning that December to February malaria incidence can be partly predicted from seasonal climate forecasting methodologies. An association between mean annual temperature and malaria prevalence was also observed in Botswana [29]. In contrast a study in Ngamiland District in Botswana did not consider temperature to be a major limiting factor in malaria transmission [30].

Relationships between rainfall and malaria transmission have been reported in many studies worldwide [31–33]. In Botswana an above average rainfall season of 1992–1993 following the 1991–1992 devastating drought was associated with a very high malaria season in 1993 [28]. Similarly Craig et al., [29] found a significant association between summer rainfall and malaria prevalence in Botswana. However the December to February 2000 rainfall was the highest and yet Botswana experienced lower than expected malaria incidence during that period [28]. In Ngamiland District, Botswana, transmission of malaria was shown to follow the rainfall pattern with a lag period of 3 months and there was some association with water discharge at Mohembo [30].

Floods indirectly lead to an increase in malaria through the expansion in the number and range of vector habitats that enhance the potential for exposure to infection by both disaster- affected population and emergency workers. The intensity of malaria transmission is further compounded by other risk factors such as changes in human behaviour due to displacement, overcrowding in temporary shelters, poor environmental conditions created by floods, post-flood destruction of the living environment, in some cases lowered physical strength due to shortage of food and creation of an environment favouring propagation of malaria infected mosquitoes, among other factors [9]. Malaria epidemics attributed to flooding have been reported in many endemic countries such as Mozambique [34], Sudan [35] and Dominican Republic [9]. However, the effect of flooding on malaria transmission was not established in Honduras following Hurricane Mitch [36].

In Botswana only three studies [28–30] have been conducted using different approaches in analysing some of the climatic variables. In view of the uncertainties regarding the effects of worldwide climate change on malaria and conflicting findings from previous reports, this study was conducted in Botswana to verify some of the previous findings on temperature and rainfall relationships with regard to malaria transmission and exploit the possibility of using flood discharge and flood extent in explaining trends on the incidence of clinical malaria cases as this had not been exhaustively done before in Botswana. Our main focus was on the relationship between the incidence of clinical malaria cases and climatic factors (rainfall, temperature, flood discharge and flood extent at micro-level) in Tubu village, Okavango sub-district in Botswana.

## Materials and Methods

### Country profile

Botswana, a landlocked country in southern Africa, has a total population of 2 024 904 [37]. Annual rainfall for Botswana is low and has a high spatial and temporal variability [38]. More than 90% of rainfall is in summer months from October to April with the annual average ranging from 250 mm in the southwest to 650 mm in the northwest part of the country [38]. The average annual temperature is about 21.2°C, with an average maximum of 32°C being experienced in October to November and average minimum of 6°C in June to July [38]. The Okavango River originates from the Angola highlands and ends in the Okavango Delta, or during wet periods in the large Makgaladgadi saltpans in the Kalahari [39]. Flooding in the Okavango Delta system varies seasonally, from year to year and from decade to decade [39]. There is another localised wet period caused by rains occurring from December to March (Ramberg et al., 2006). However, during years of heavy rainfall over the delta extensive flooding can occur in January and continue until the second flood peak that occurs between April and July [39]. Malaria is distributed in the northern half of the country (Okavango sub-district) in which our study village is located.

Although the country profile is designated as epidemic for malaria, parts of Okavango sub-district are endemic. This is primarily attributed to persistent swamps within the Okavango Delta area. According to the Ministry of Health [40]since 2005 reported annual malaria cases in the Okavango sub-district have been fluctuating with unconfirmed cases ranging from 4 686 in 2005 to 10993 in 2006 and confirmed cases ranging from 5 (in 2012) to 791 (in 2006). The maximum number of deaths recorded during the same period (2005 to 2012) was 16 and it occurred in 2006, indicating that 2006 was the worst since 2005.

### Study village

**Fig 1:**
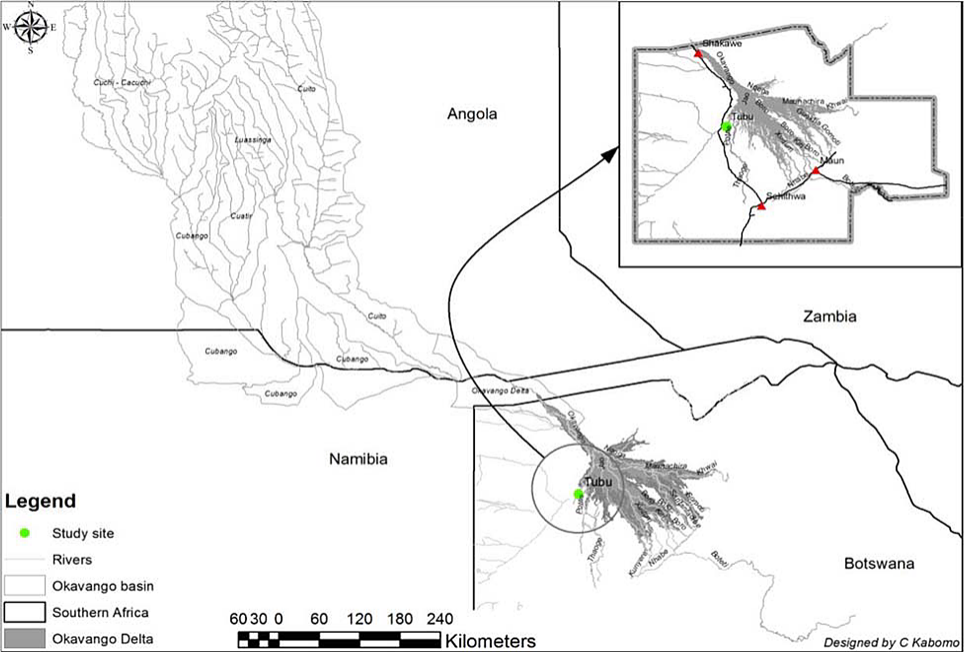
Location of Tubu village in the Okavango Delta.

Our study was conducted in Tubu village in the Okavango sub-district. The village is on the banks of the Thaoge River (Fig 1), one of the distributaries of the Okavango Delta and stands at an altitude of about 950 m above sea level and between latitude 19°35^/^S and longitude 22°27^/^E with a total population of 483 [37]. Flooding in the study village occurs every year but the extent of flooding varies. The village is serviced by a single Health Post (clinic). The nearest other health facility (Gumare District Hospital) is 10 km from Tubu Health Post.

### Data collection

The study was approved by the Health Research and Development Committee of the Ministry of Health, Botswana (Ethical Clearance Number:-PPMC:13/18/1 Vol VIII (32) of 2013). However the community was verbally informed of the objectives of our study during a general sensitization meeting and unanimously approved our study. Verbal informed consent was sought from the community since the data from the clinic was de-identified before extracting from Health Post records. The malaria clinical data obtained from Health Post records (T5 forms) was anonymized since it did not indicate patients’ identities.

Health Post data (data in S1 Dataset) on clinical malaria cases was collected retrospectively for the period July 2005 to June 2010 at Tubu Health Post. Data sets for the period after October 2010 were not available due to changes in Government policy on the recording of clinical and confirmed malaria cases at all Health Posts in Botswana. The out-patients register was used to extract information on the number of new malaria cases per month and per year in each age group. The information in the register was already aggregated by the Health Post personnel to obtain the total number of malaria cases per month in each of the three age categories. Besides malaria the register captured information on the number of patients presenting with any other diseases. The three categories for age groups, as reflected in the register, were <1year, 1 to 4 years and above 5 years. Since our study area is believed to be having low malaria endemicity due to the elimination programme diagnostic algorithms for a clinical malaria case at the Health Post were a fever (with a temperature above 37.5°C), in the absence of measles, running nose or other cause of fever, as for children as described by the WHO (2005). For adults it was intermittent fever (with a temperature above 37.5°C) accompanied by at least headache, vomiting or reduced feeding.

Daily temperature figures specifically for Tubu village were obtained from the district office at Shakawe Meteorology Station. Shakawe Meteorology Station represented the central district office where all records from all meteorology stations in all district villages were kept. The figures were converted into mean monthly minimum, mean monthly maximum and mean average temperatures (data in S2 Dataset). Monthly rainfall data for Tubu village was retrieved from recordings done by Gumare Agricultural Research Station (data in S4 Dataset). Information on monthly flood discharge, measured at Mohembo site, was obtained from the Department of Water Affairs (data in S5 Dataset). Data for maximum monthly flood extent (area) was derived from 8 day composite sets of Moderate Resolution Imaging Spectroradiometer (MODIS) imagery with a spatial resolution of 250 km done by the Okavango Research Institute, University of Botswana (data in S3 Dataset).

### Data analysis

Number of new clinical malaria cases recorded per month in the clinic register were converted to incidence rates of clinical malaria cases and presented as the number of new malaria cases per month and per year per every 1000 individuals at risk of malaria in the village and used as an indicator for malaria transmission. The incidence rates for clinical malaria cases were correlated to climatic indices:- monthly temperature (°C), monthly rainfall (mm), maximum monthly flood extent (km^2^) and monthly flood discharge (Mm^3^/month). The SPSS version 21.0 statistical package was used to generate graphs and to perform the normality and one-way analysis of variance (ANOVA) tests. The Pearson correlation coefficient test was run using the Stata 13 Software. Appropriate graphs were generated to show trends on climatic factors and clinical malaria incidence rates over the study period. The assumption for normality was tested using the Shapiro-Wilk test at the 0.001 level of significance on annual data for incidence rates on clinical malaria cases. Since the *p*-values were all greater than 0.001, it was concluded that each of the independent variables were normally distributed. The one-way analysis of variance (ANOVA) was used to determine if there was any significant difference in annual incidence rates for clinical malaria among the different age groups. A Pearson correlation coefficient (rho [ρ]) was run to determine the relationship between the incidence of clinical malaria cases and climatic variables. Lagging of climatic variables was done for 0 to 6 months in order to determine maximum significant positive cross-correlations.

## Results

### Trends on the incidence of clinical malaria cases

**Fig 2:**
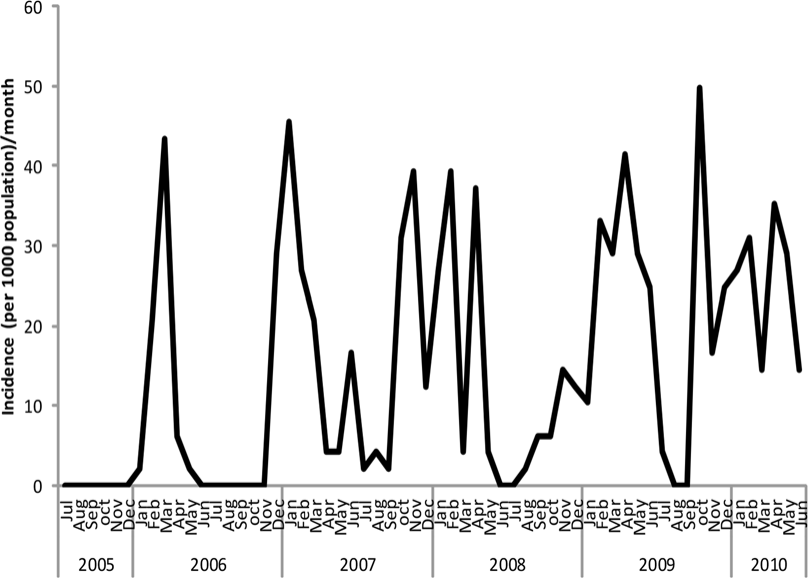
Trends on incidence rates of clinical malaria cases reported at Tubu Health Post.

Incidence of clinical malaria cases was reported almost every month of each year with distinct peak periods for different seasons (Fig 2). The highest incidence rate per 1000 population at risk of malaria was 49.7, recorded in October 2009 (Fig 2). Generally the peak periods were recorded between October and May.

**Fig 3:**
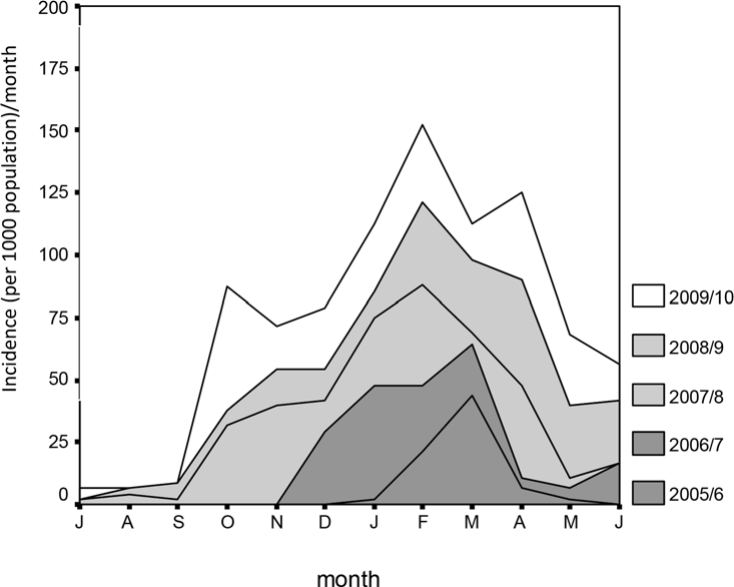
Cumulative incidence rates of clinical malaria cases reported at Tubu Health Post.

The highest cumulative incidence of clinical malaria cases for the period July 2005 to June 2010 was in February (Fig 3).

**Fig 4:**
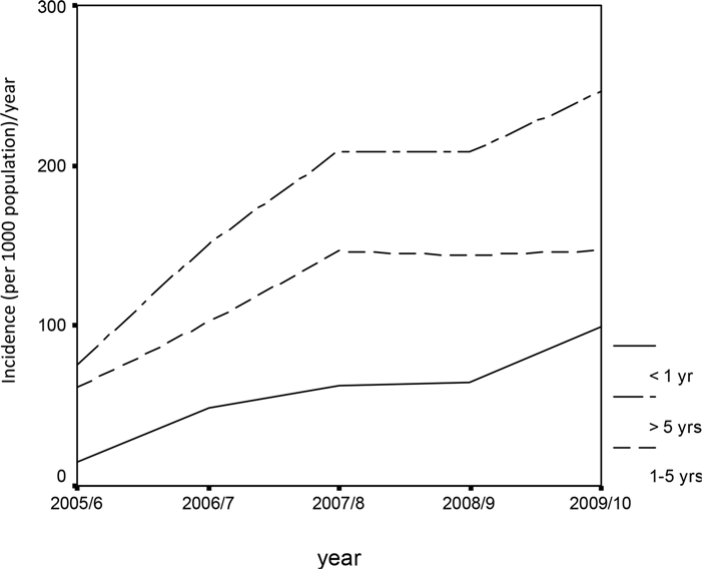
Age pattern of the incidence of clinical malaria cases at Tubu Health Post.

There was a sharp increase in annual incidence rate of clinical malaria cases between July 2005 and June 2007 followed by a decline recorded from July 2007 to June 2008 (Fig 4). Thereafter there was a slight increase recorded between July 2008 and June 2010.

There was a significant difference in the incidence of annual malaria cases among the three age categories of <1 yr, 1–5yrs and >5yrs (Univariate ANOVA: p < 0.001). The difference was highly significant between the age groups >5yrs and 1yrs (p<0.001) and age groups >5yrs and 1–5yrs (p<0.001). There was no significant difference between age groups <1yr and 1–5yrs (p>0.05). Incidence rates for the age group < 1yr and 1–5yrs were pooled together to give a single age group of >0–5 yrs. Highest incidence rates were reported from the age group > 5 yrs than >0–5 yrs (Fig 4).

### Incidence rates versus un-lagged variables

**Fig 5:**
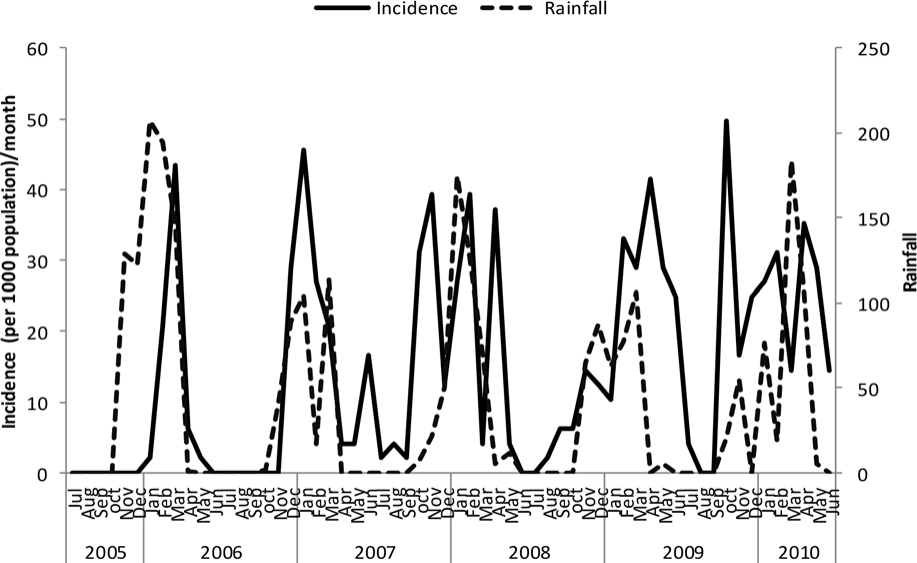
Relationship between incidence of clinical malaria and rainfall in Tubu village.

**Fig 6:**
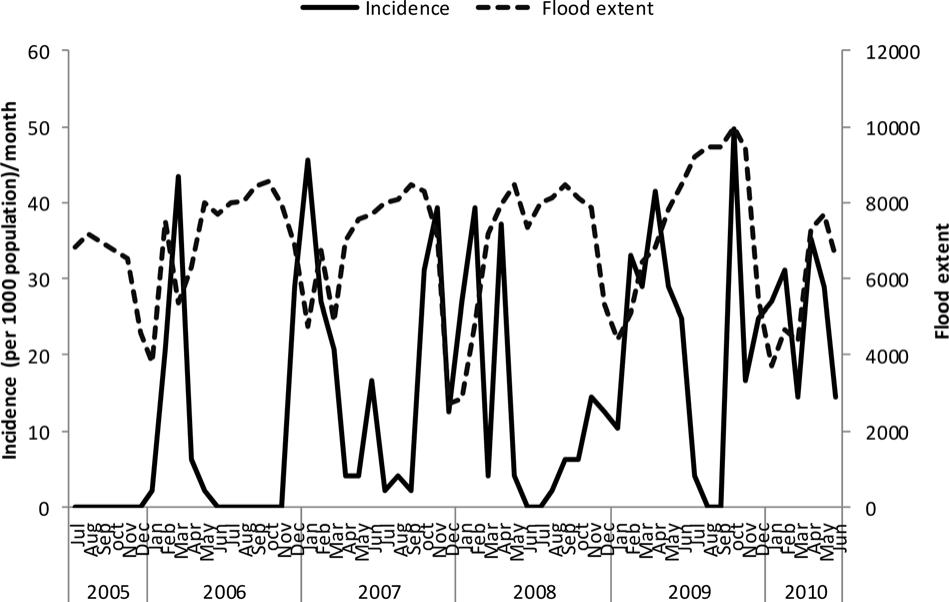
Relationship between incidence of clinical malaria and flood discharge in Tubu village.

**Fig 7:**
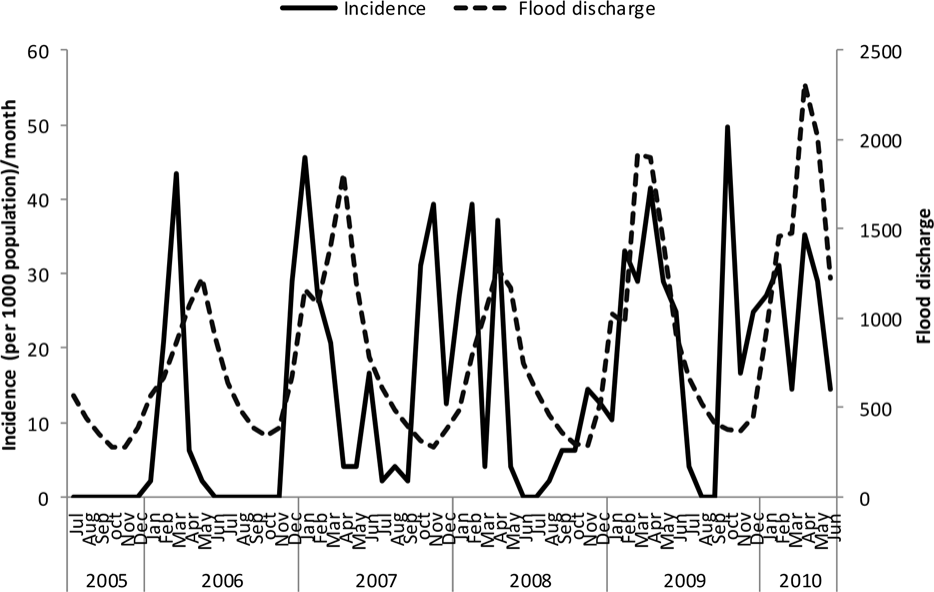
Relationship between incidence of clinical malaria and mean minimum temperature in Tubu village.

**Fig 8:**
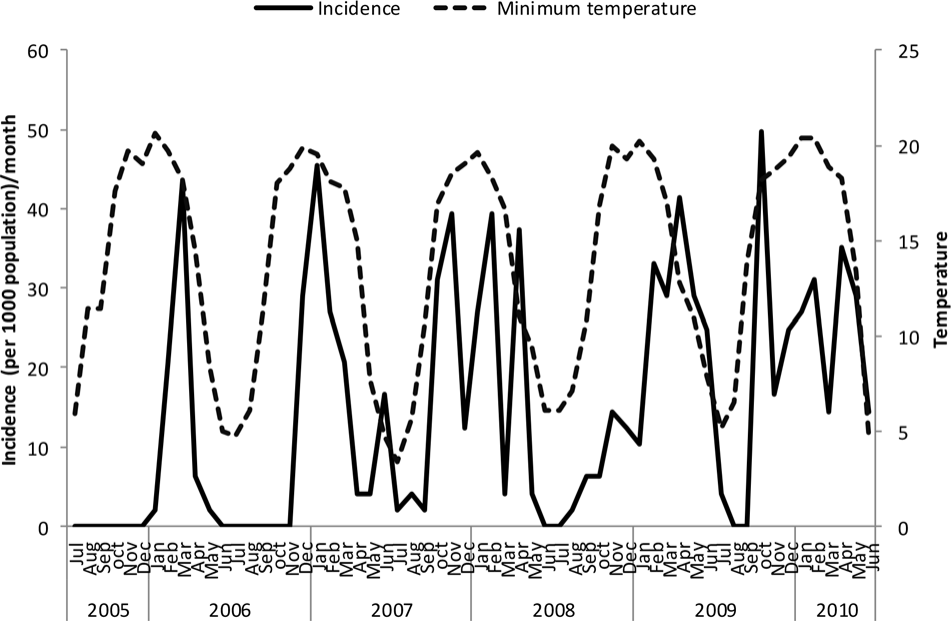
Relationship between incidence of clinical malaria and mean maximum temperature in Tubu village.

**Fig 9:**
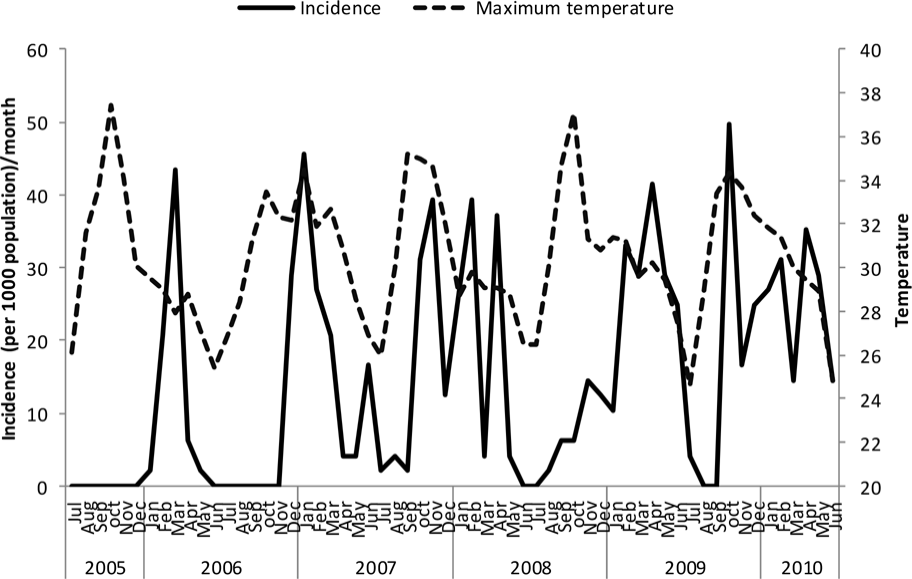
Relationship between incidence of clinical malaria and average temperature in Tubu village.

**Fig 10:**
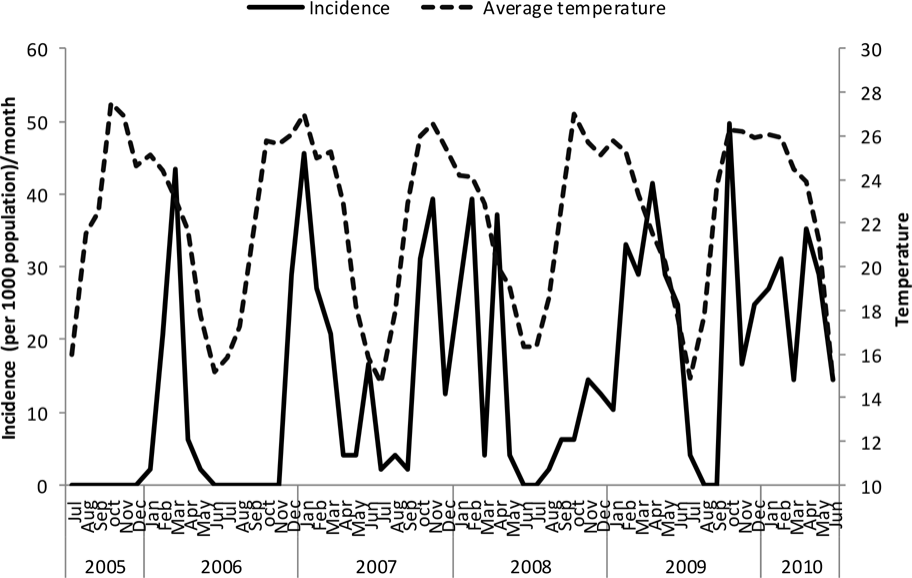
Relationship between incidence of clinical malaria and flood extent in Tubu village

Un-lagged graphical presentations showed some form of relationship between the incidence rates and rainfall (Fig 5), discharge (Fig 6) and temperature (Figs 7–9) but none with flood extent (Fig 10). The graphs (Figs 5–10) show that climatic variables and the incidence rates follow the same pattern although at different peak periods.

### Incidence rates versus lagged variables

Strong correlations were observed between the incidence of clinical malaria and climatic variables at different monthly lag periods (Table 1).

**Table 1:** Pearson correlation coefficients between incidence of clinical malaria cases and lagged climatic variables.

	Pearson correlation coefficient (rho [ρ])					
Malaria incidence versus:	Rainfall	Flood extent	Discharge	Temperature (average)	Temperature (minimum)	Temperature (maximum)
Zero lag	0.299*	-0.257*	0.396*	0.385*	0.452*	0.151
Lag (1 month)	0.417*	-0.252	0.177	0.493*	0.537*	0.277*
Lag (2 month)	0.250	-0.255*	-0.044	0.445*	0.442*	0.328*
Lag (3 month)	0.129	-0.040	-0.247*	0.290*	0.294*	0.203
Lag (4 month)	-0.175	0.129	-0.359*	0.154	0.073	0.269*
Lag (5 month)	-0.368*	0.354*	-0.281*	-0.044	-0.192	0.248
Lag (6 month)	-0.462*	0.467*	-0.077	-0.276*	-0.392*	0.018

**Bold:** maximum positive correlation

*: significant correlation (at the 5% level)

A positive correlation was strongest against flood discharge at zero-month lag period. However negative correlations were observed from the second to the sixth month lag period. Average temperature, minimum temperature and rainfall were highly positively correlated with incidence of clinical malaria cases at 1-month lag periods. Negative correlations between rainfall and incidence of clinical malaria cases were observed from the fourth to the sixth month lag period. The maximum significant positive correlation between maximum temperature and incidence of clinical malaria cases was observed at 2-month lag period. The maximum significant positive correlation between flood extent and incidence of clinical malaria cases was observed at 6-month lag period.

## Discussion

The highest number of malaria cases recorded in Tubu village were between October and May with varying seasonal peak periods. Peak malaria seasons vary from country to country within southern Africa but are generally highest during and after the rainy seasons (November to May, peaking between February and April). The peak malaria transmission season in Tubu village is within the range of peak periods observed in Zimbabwe [41], Mpumalanga, South Africa and Maputo [42]. The trend in overall incidence of malaria cases from 2005/2006 to 2009/2010 malaria seasons may be attributed to other factors beyond the scope of this study.

Malaria cases were mainly in the older age group (>5yrs) throughout the five consecutive years. This confirms findings from a previous study conducted in Ngamiland district, Botswana where both confirmed and unconfirmed cases were higher in age group >5yrs [30]. This is an indication of very low transmission in Tubu village because ordinarily, in a high malaria endemic area, <5’s are usually the most affected [43]. The Ministry of Health [40], Botswana has reported to have made great strides in the elimination of malaria. It has been noted that declines in malaria transmission intensity and progression to malaria elimination results in the burden of malaria shifting towards older children [44–46], hence more cases of malaria being reported among 5yrs and above in Tubu village. A study in north-east Tanzania also provided evidence of a shift in malaria intensity from younger children to older children due to a decline in malaria transmission and prevalence through control efforts [47]. Our findings indicate that the Botswana’s Ministry of Health (MoH) malaria control interventions may be effective.

We established positive correlations between malaria cases and minimum and average temperatures at 1-month lag periods. Minimum temperature plays an important role in enhancing survival chances of vector mosquitoes and associated *Plasmodium* parasites during winter and by so doing accelerates the transmission of malaria [26]. Stronger and significant associations between the incidence of clinical malaria cases and minimum and average temperatures at 1-month lag periods indicate that minimum and average rather than maximum temperatures play important roles in malaria transmission in Tubu village. The same observations were made in other regions of the world including Rwanda [26], Shuchen County, China [48], East African Highlands [19, 49], where minimum temperature played a significant role in malaria transmission at 1-month lag periods. Very low correlation coefficient on incidence of clinical malaria cases versus maximum temperatures compared to minimum temperatures for the 1-month to 3-month lag periods suggest that maximum temperature was not a significant factor in the transmission of malaria in Tubu village. Since the effect of maximum temperature was strongest at 2-month lag period, its contribution to the biological cycle of malaria was therefore relatively insignificant. Maximum temperature was also considered to be of less significance in predicting *Plasmodium falciparum* malaria in climatic zones of Ethiopia [50]. Similarly, in Ngamiland district, Botswana temperature did not appear to be a major limiting factor in malaria transmission as average annual temperatures did not drop below the critical temperature (for malaria parasite development in the mosquito) of 18°C for prolonged periods [30].

In our study a 1-month lag period on the association between the incidence of malaria cases and rainfall seems to be the ideal time frame for the biological pathway of *P*. *falciparum* malaria transmission [48]. Our findings corroborate with those of other studies conducted in Mpumalanga, South Africa [42], Shuchen County, China [48], The Gambia [51] and Sri Lanka [52]. Other studies that have found a link between rainfall and malaria cases were conducted in South Africa [53], Ethiopia [50], Zimbabwe [54], Sri Lanka [55] and Republic of Korea [17]. In Botswana positive correlations between malaria and summer rainfall [29] and malaria and heavy rainfall [28], were reported. A similar study by Chimbari [30], showed some correlation between malaria transmission and rainfall pattern with however an unusual lag period of three months.

The relationship between the incidence of clinical malaria cases and flood extent and discharge is a complex one. There was a lag period of 6 months between onset of malaria cases and flood extent and no lag period between onset of malaria cases and flood discharge. Tubu village is at the lower end of the Okavango Delta which receives flood waters between the months of June/July and recede from August onwards, declining to their lowest level in November. This means maximum flood extent is experienced 6 to 7 months post-discharge. The maximum and significant positive correlation between malaria cases and flood extent was at 6-months lag period, which translates to around January/February of the following year. Therefore, maximum contribution of floods to malaria transmission is realised mostly in the months of January and February when the floods have receded and left behind breeding habitats. Floods may initially flush out mosquito larvae, but many breeding places emerge when the water recedes; the lag time after receding being usually around 6–8 weeks before the onset of a malaria epidemic [9]. In Tubu the receding floods create much year-round breeding habitats for mosquitoes and the habitats continue to be sustained by rain when it falls around October [56]. In addition to heavy rainfall over the delta, extensive flooding can already occur in January and continue until the second flood peak appears between April and July [39], thus providing year-round mosquito breeding habitats, that result in malaria transmission occurring throughout the year. Since flood extent is directly related to amount of discharge it follows that both are correlated to the incidence of clinical malaria cases. An association between flood discharge at Mohembo and malaria cases in Ngamiland district, Botswana was also reported [30].

### Limitations of the study

Analysis was based on routinely collected clinical malaria data from Tubu Health Post. There is a possibility of both under and over reporting of malaria cases due to other febrile illnesses which mimic with malaria. However personnel at Health Post were well trained and regularly received refresher courses on clinical diagnosis of malaria cases. Since retrospective data were used its accuracy and completeness could not be fully verified. Most of the cases reported at the clinic were not confirmed by the Rapid Diagnostic Tests (RDT) and microscopy due to resource limitations but were based on clinical signs and symptoms and this could also contribute to some potential for misclassification. However, a previous study by Thomson et al. [28] in Botswana showed that data sets for unconfirmed and confirmed malaria incidence were highly correlated. A study in Zimbabwe also confirms that although clinical cases may be far more than the actual cases as confirmed by microscopy, the trend of clinical cases in an area is generally similar to that of microscopically confirmed cases [57]. More information could have been obtained on malaria trends from 2005 to 2013 if the MoH had not changed its policy on the malaria case reporting system at all Health Posts in Botswana with effect from October 2010. The Ministry of Health, Botswana, policy stipulates that all suspected malaria cases should be referred to District Hospital for confirmation and unfortunately there is no feedback to the Health Posts on the outcome of the patient at the District Hospital. Furthermore, consistent information on confirmed clinical malaria cases from Tubu Health Post was not easily accessible for various reasons. Hence, the use of clinical malaria cases (unconfirmed) data from the Health Post for our analysis.

## Conclusions

Malaria transmission pattern in Tubu village followed the trends observed in other southern African countries. However peak periods varied from year to year. The age group >5 years was identified as experiencing the greatest burden of clinical malaria. The correlation between climatic factors and incidence rates of clinical malaria cases was identified. Flood discharge and extent, rainfall, minimum and average temperatures were found to be associated with the incidence of clinical malaria cases. Maximum temperature was not associated with the incidence of clinical malaria cases.

### Recommendations

Since some correlations were observed between climatic variables and malaria cases some predictive models can be applied to determine if these factors could accurately predict malaria transmission levels. Management of confirmed malaria cases should be re-introduced at Health Posts as a foundation for further studies, monitoring of the impact of intervention strategies and for forecasting of disease trends using climatic variables at micro-level. Malaria transmission is very complicated, and for a better understanding of the links between climatic variables and malaria transmission and formulation of predictive models, additional detailed information that is important includes: high quality time series entomological data, malaria control interventions, malaria epidemiology and ecological/environmental factors and health outcomes (eg. date of onset, site of onset, etc).

## Supporting Information

S1 Dataset Incidence rates (per 1000) of clinical malaria cases (per month and per age-group) reported at Tubu Health Post. (XLSX)

S2 Dataset Minimum and maximum temperature (°C) recordings in Tubu village. (XLSX)

S3 Dataset Monthly flood extent (km^2^) measured in Tubu village. (DOC)

S4 Dataset Monthly rainfall (mm) figures recorded in Tubu village. (DOC)

S5 Dataset Monthly flood discharge (Mm^3^/month) recorded at Mohembo. (XLSX)
