# Supplementary material for: Clinical Malaria Transmission Trends and Its Association with Climatic Variables in Tubu Village, Botswana: A Retrospective Analysis
Source: PLoS One. 2016 Mar 16;11(3):e0139843. doi: 10.1371/journal.pone.0139843 (PMC4794139; doi:10.1371/journal.pone.0139843)
Supplement: S3 Dataset — (DOC) [file pone.0139843.s003.doc]

**S3 DATASET. Monthly flood extent (km^2^) measured in Tubu village**

| **MONTH** | | | **2004/05** | **2005/06** | **2006/07** | **2007/08** | **2008/09** | **2009/10** | **2010/11** |
| --- | --- | --- | --- | --- | --- | --- | --- | --- | --- |
| **J** | | | 7895.438 | 6837.813 | 7998.375 | 8012.438 | 8004.688 | 9216.250 | 10101.310 |
| **A** | | | 7982.250 | 7192.438 | 8055.000 | 8101.500 | 8106.938 | 9470.000 | 10142.500 |
| **S** | | | 7988.000 | 6965.875 | 8436.875 | 8451.875 | 8478.750 | 9485.250 | 10453.190 |
| **O** | | | 7877.125 | 6724.188 | 8557.938 | 8288.000 | 8144.125 | 10003.500 | 10315.250 |
| **N** | | | 6902.875 | 6523.375 | 7937.250 | 7151.250 | 7850.313 | 9415.563 | 5761.500 |
| **D** | | | 7354.750 | 4588.438 | 6865.500 | 2736.500 | 5327.188 | 5451.250 | 6147.188 |
| **J** | | | 6356.188 | 3800.000 | 4761.188 | 2859.875 | 4408.813 | 3712.875 | 2619.313 |
| **F** | | | 5083.875 | 7537.875 | 6746.625 | 4844.563 | 5098.750 | 4651.125 | 5405.500 |
| **M** | | | 5919.500 | 5357.000 | 4858.875 | 7159.750 | 6449.875 | 4364.188 | 6224.313 |
| **A** | | | 6949.688 | 6298.938 | 7014.375 | 7970.500 | 6815.063 | 7292.750 | 5584.750 |
| **M** | | | 5832.938 | 7981.813 | 7546.750 | 8490.000 | 7823.875 | 7673.375 | 5135.938 |
| **J** | | | 6653.563 | 7699.375 | 7693.000 | 7345.625 | 8477.250 | 6574.875 | 10553.130 |
| **Annual**  **Average** | | | **6899.683** | **6458.927** | **7205.979** | **6784.323** | **7082.136** | **7275.917** | **7370.324** |
|  |  |  | | | | | | | |
|  |  |  | | | | | | | |
