# Supplementary material for: Clinical Malaria Transmission Trends and Its Association with Climatic Variables in Tubu Village, Botswana: A Retrospective Analysis
Source: PLoS One. 2016 Mar 16;11(3):e0139843. doi: 10.1371/journal.pone.0139843 (PMC4794139; doi:10.1371/journal.pone.0139843)
Supplement: S4 Dataset — (DOC) [file pone.0139843.s004.doc]

**S4 DATASET. Monthly rainfall (mm) figures recorded in Tubu village**

| **Year** | **Mean monthly rainfall (mm)** | | | | | | | | | | | | **Annual R/F (mm)** |
| --- | --- | --- | --- | --- | --- | --- | --- | --- | --- | --- | --- | --- | --- |
|  | **J** | **A** | **S** | **O** | **N** | **D** | **J** | **F** | **M** | **A** | **M** | **J** |  |
| **2005/06** | 0.0 | 0.0 | 0.0 | 0.0 | 128.5 | 122.8 | 207.0 | 194.6 | 146.1 | 1.0 | 0.0 | 0.0 | 800.0 |
| **2006/07** | 0.0 | 0.0 | 0.0 | 1.8 | 40.0 | 89.1 | 104.5 | 17.1 | 113.4 | 0.0 | 0.0 | 0.0 | 365.9 |
| **2007/08** | 0.0 | 0.0 | 0.0 | 6.7 | 21.3 | 50.5 | 174.4 | 125.9 | 69.8 | 5.0 | 12.0 | 0.0 | 465.6 |
| **2008/09** | 0.0 | 0.0 | 0.0 | 0.0 | 64.6 | 87.4 | 63.2 | 76.9 | 105.8 | 0.0 | 5.0 | 0.0 | 402.9 |
| **2009/10** | 0.0 | 0.0 | 0.0 | 20.3 | 54.5 | 0.0 | 76.3 | 18.5 | 183.5 | 106.2 | 5.0 | 0.0 | 464.3 |
| **2010/11** | 0.0 | 0.0 | 0.0 | 0.0 | 89.0 | 32.0 | 203.3 | 67.0 | 72.8 | 93.7 | 6.0 | 0.0 | 563.8 |
| **2011/12** | 0.0 | 0.0 | 0.0 | 0.0 | 73.7 | 68.1 | 46.5 | 109.0 | 74.5 | 0.0 | 0.0 | 0.0 | 371.8 |
